# Risky sexual practice and associated factors among HIV positive adults visiting ART clinics in public hospitals in Addis Ababa city, Ethiopia: a cross sectional study

**DOI:** 10.1186/s12889-019-6438-5

**Published:** 2019-01-28

**Authors:** Wondimagegne Belay Tadesse, Abebaw Addis Gelagay

**Affiliations:** 1 EPHIA project, Addis Ababa, Ethiopia; 2 Department of Reproductive Health, College of Medicine and Health Sciences, University of Gondar, Gondar, Ethiopia

**Keywords:** Risky sexual practice, HIV positive adults, Visiting ART clinic, Ethiopia

## Abstract

**Background:**

Sexual behavior of HIV positive individuals visiting anti- retroviral clinics is a neglected issue. With access to anti-retroviral treatment, HIV positive individuals experience improved health and are able to reintegrate into their social life and many of them engage in sexual activities. In the context of Ethiopia, safer sex practices among people living with HIV is critical in terms of preventing the acquisition of another strain of HIV and helping address the epidemic.

**Method:**

An institution-based cross sectional study was conducted at Addis Ababa public hospitals from January to February 2017. A pretested structured questionnaire was used to collect the data. Using the systematic random sampling technique, a total of 562 respondents participated in the study. The data were entered into EPI info version 3.5.3 and transferred to SPSS version 20 for analysis. Descriptive statistics, bi-variate, and multi variable analyses were done. A *p*-value < 0.05 was considered to determine the statistical significance of the association between factors (independent variables) and risky sexual practice. The Odds ratio was also used to determine the presence and the degree of association between dependent and independent variables.

**Results:**

A total of 562 respondents participated in the study which revealed that the prevalence of risky sexual practice was 39.1% (95% CI: 35.2, 43.8) three months prior to the data collection. Educational status of participants who were below grade eight (AOR = 2.27, 95% CI:1.01,5.10) and went to grades eight to twelve (AOR = 2.12, 95% CI:1.02,4.41), were married (AOR = 2.07, 95% CI:1.06,4.02), had no concern for safer sexual practice (AOR = 3.74, 95% CI:2.28, 6.13), had CD_4_ count of ≥500cells/mm^3^(AOR = 1.66, 95% CI:1.04, 2.64), and used substance (AOR = 3.41, 95% CI:1.83, 6.35) were significantly associated with risky sexual practice.

**Conclusion:**

The prevalence of risky sexual practice was markedly high in this study due to such contributory factors as low educational status, marriage, lack of concern for safer sexual practices, and substance use.

**Electronic supplementary material:**

The online version of this article (10.1186/s12889-019-6438-5) contains supplementary material, which is available to authorized users.

## Background

Like the other Sub-Saharan African countries, which contribute two-thirds of the new global HIV infection, Ethiopia is worst hit by the HIV/AIDS pandemic [1]. According to HIV-related estimates and projections for Ethiopia, the adult HIV prevalence was estimated at 1.2% (0.8% in males and 1.6% in females); such incidence stood at 0.03% in 2014. The mortality and incidence rates have dropped by about two-thirds since the initiation of ART the Program in 2005 [2]. Although the incidence and prevalence rates have shown a declining trend, there are more than 724,400 people living with HIV/AIDS in Ethiopia today [3].

HIV infected individuals show good physical improvement and feel well after they start to use antiretroviral drugs [4].This general improvement in their physical condition and feeling of well-being lead most of them to reintegrate into social life, and many engage in sexual activities. Since they are on ART, most of the HIV positive individuals believe that they are not at risk of transmitting the virus [5]. But one thing that should be taken into consideration is that if they practice unprotected sex which while their viral loads are detectable not only do they have the potential to transmit the HIV virus and other STI’s but also acquire other strains of the virus which can’t be suppressed with the current ART drug they are taking [6].

The available studies show contradicting ideas on the risky sexual practices of HIV positive individuals who are on ART. Some studies suggest risky sexual practice decreased among individuals on ART because of the intensive counseling they were getting during their follow ups; others argued that it showed increases after the initiation of ART because of the physical well-being that followed ART initiation; still others concluded ART has no effect on it [3].

Adopting safer sex practices regardless of the use of ART is important for avoiding HIV transmission as many persons living with HIV in developed countries [7]. The use of antiretroviral therapy among HIV positive individuals brings a better physical condition and feeling of well-being. However, a substantial number of HIV positive individuals engage in risky sexual practices which the few available studies are unable. Therefore, this study aimed to determine the magnitude and factors associated with it by using a comprehensive definition for risky sexual practice.

## Methods

### Study setting

A cross-sectional study was conducted on HIV positive adults visiting ART clinics in Addis Ababa from January to February 2017. According to the 2015 Ethiopian sub-national HIV estimate, HIV prevalence in the city was 4.2%, with a total of about 116,800 people infected [2]. There were 53 public and private ART-providing health institutions in Addis Ababa as per the November 2016 report, of which 9 were public hospitals, 23 were public health centers and the rest were public clinics. Overall, about 17,068 People Living with HIV AIDS (PLWHA) were on ART at the time of the study.

### Study population

The study participants were all HIV positive adults over 18 years of age who tested three months prior to the data collection. Those who made two or more clinic visits were included in the study, while participants who were sick and unable to communicate were excluded from the study.

### Sample size and sampling technique

Sample size calculation was done using the single population formula by taking the prevalence of (p) 30.4% from a previous study [5] and by considering a 95% CI, 80% power, 4% marginal error, and a 10% non-response rate. The sample size 562, was allocated proportionally to the number of clients on ART at each hospital. All public hospitals under the Addis Ababa Health Bureau administration were included in the study. Registered adult clients on ARTat Gandi Memorial, Minillik II, Ras Desta, Tirunesh Bejing, Yekatit 12, and Zewditu Memorial hospitals were 954, 2503, 2200, 279, 3600, 7532, respectively. The systematic random sampling technique was used at hospital level.

### Data collection technique

A structured pre-tested interviewer administered questionnaire was used for data collection (attached as Additional file 1). The questionnaire was developed by reviewing national and international literature. The data collection instrument was prepared in English and was translated first to Amharic and back to English in order to ensure consistency. The questionnaire contained information on socio-demographic characteristics, and on factors such as relationship, medical, psycho-social, and behavioral.

Training was given to data collectors and supervisors on the objectives of the work and the data collection tools; then, the tools were pre-tested on 5% of the total sample to check their clarity. Items of the questionnaire found difficult in the pre-test were rephrased and corrected. Data were collected by twelve diploma graduate nurses (male data collectors for male respondents and females for females) who were working in the ART clinics of the respective hospitals. The data collection took place at the ART clinics of each hospital. The principal investigator and two supervisors monitored the data collection process, and checked the completeness and consistency of the questions.

### Measurements

The main outcome variable for the study was risky sexual practice. The independent variables were socio-demographic characteristics including sex, age, ethnicity, educational status, religion, marital status, occupation and monthly income status; relationship factors like discussion about safe sex, partner HIV status and disclosure status; medical related factors such as duration of diagnosis of HIV, time lapse after the start of ART and CD_4_ count; behavioral factors, including general self-efficacy, active substance use, and sex under the influence of alcohol; psycho-social factors, such as stigma and depression.

#### Stigma

In this study, stigma was assessed by eight questions addressing enacted stigma encountered since testing positive and six questions addressing perceived stigma any related to avoidance, social rejection, and shame in 3 months prior to the study with Yes/No responses [8].’Yes’ was coded as (1) and ‘No’ as (0). A score above the mean of the total responses was considered as stigmatized or felt stigma.

#### Active substance use

In this study, use of active substances was defined as taking any sort of stimulants, which alters the body physiology; E.g. khat, shisha, hashish (marijuana), cocaine, benzene etc. within three months prior to data collection period [6].

#### Sex under the influence of alcohol

In this study, the influence of alcohol was defined as engaging in undesirable sexual activity which was not performed at other times before taking any drinkable thing which has alcohol in it and alters body physiology, E.g. local beer, liquor, beer etc. in three months prior to date of data collection [5].

#### General self efficacy

In this study, general self efficacy was assessed by four item scales derived from the scale developed by Schwarzer and colleagues [9] that address how loyal an individual is their partner. Responses ranged from 1 (strongly disagree) to 4 (strongly agree), and a score above the mean of the total respondents was taken as high general self-efficacy.

In this study, “risk sexual practice” was defined as engaging in one of the following characteristics: sex without use of condoms or inconsistent use of condoms, multiple sexual partners, casual sex, sex with the influence of alcohol and sexual exchange (paying or receiving goods or money for sexual intercourse) within three months prior to date of data collection. *Steady partner* was a partner with whom a respondent had regular sexual relationship and perceived by them as spouse or regular boy/girl friend [6]. *Casual partners* means individuals with whom they had sexual intercourse once or a few times other than regular steady partners (spouse/boy/girl friend) on payment or no payment [6] and *Multiple sexual partners* as having sex with more than one sexual partner within three months prior to data collection period [6].

### Data analysis

The data were first entered using EPI info version 3.5.1 and then exported to SPSS version 20 for cleaning and analysis. Descriptive statistics was used to describe the socio-demographic and other characteristics of the respondents. Factors which had a potential association (*p*-value < 0.2) with the outcome variable in a bi-variate analysis were transferred to a multivariable analysis. Odds ratio with 95% CI was used to declare the presence and the strength of association in the multivariable analysis.

## Results

### Socio demographic characteristics

In this study, the response rate was 100%. All of the respondents were on ART. Most of the participants, 344(61.2%), were females with 200(35.6%) in the age group of 30–35 years, and 198(35.2%) were Amhara by ethnicity. The mean age of the participants was 33.06 ± 5.4 (SD) (Table 1).

**Table 1:** Socio demographic characteristics of respondents; visited ART clinics in Public hospitals in Addis Ababa, 2017

Characteristics	Frequency(*n* = 562)	Percentage
Sex		
Male	218	38.8
Female	344	61.2
Age		
18–23	12	2.1
24–29	153	27.2
30–35	200	35.6
36–41	162	28.8
≥ 42	35	6.2
Ethnicity		
Oromo	195	34.7
Amhara	198	35.2
Tigray	118	21.0
Others	51	9.1
Education Status		
≤ 8	218	38.8
9–12	249	44.3
College diploma and above	95	16.9
Religion		
Orthodox	342	60.9
Muslim	88	15.7
Protestant	110	19.6
Catholic	22	3.9
Marital Status		
Unmarried	211	37.5
Married	188	33.5
Separated	44	7.8
Divorced	55	9.8
Widowed	64	11.4
Occupation		
Unemployed	22	3.9
NGO	40	7.1
Daily laboror	88	15.7
House wife	99	17.6
Government employee	110	19.6
Private job	203	36.1
Monthly Income		
< 1500	162	28.8
1500–2999	262	46.6
≥ 3000	138	24.6

### Prevalence of risky sexual practice

In this study, 39.1% (95%CI: 35.2, 43.8) of the participants had risky sexual practice, i.e., unprotected sex in the last three months prior to data collection. Of all participants, 317(56.4%) were sexually active and 77(24.3%) of whom had more than one sexual partners. Well over two-third (225), 60, and 32 of these sexually active respondents had steady, casual, and both steady and casual partners respectively. Among the sexually active study participants, 175(55.2%) used condoms in the past three months prior to data collection 123(70.3%) of whom used condoms consistently, while the rest used it inconsistently or didn’t use at all. Some of the reasons mentioned by participants for not using condoms were both partners were HIV positive and that condoms decrease sexual pleasure. Besides, partners didn’t want to use condoms because they wanted to get children and for religious reasons (Table 2).

**Table 2:** Partner related characteristics, partners HIV status and disclosure among HIV positive adults visited ART clinics in Addis Ababa public hospitals, 2017

Characteristics	Frequency	Percentage
Had sexual partner in the past three months		
Yes	317	56.4
No	245	43.6
Number of sexual partner/s in the past three months(*n* = 317)		
One	240	75.7
More than one	77	24.3
Type of sexual partner/s have in the past three months(*n* = 317)		
Steady	225	71
Casual	60	18.9
Both	32	10.1
HIV status of their partner/s(n = 317)		
Positive	173	54.3
Negative	10	3.1
Both	25	8.4
Unknown	109	34.2
Discussion about safe sex with their partner/s(n = 317)		
Yes	129	40.69
No	188	59.31
HIV status disclosure to partner(n = 317)		
Yes	182	57.41
No	135	42.59

**Fig. 1:**
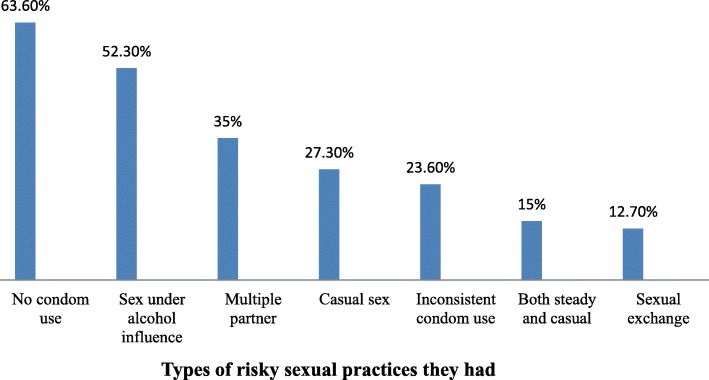
Figure that shows the types of risky sexual practice that the participants had

Among the sexually active participants, 69.4% had risky sexual practices. Among participants who had risky sexual practice, most (63.6%) had sex without condom and a few (12.3%) engaged in sexual exchange three months prior to data collection (Fig. 1).

### Factors associated with risky sexual practice

Each variable was analyzed using bivariate logistic regression, and variables with less than 0.2 were fitted to the multivariable logistic regression. In the multivariable analysis, educational status (*p* < 0.05), marital status (*p* = 0.03), concern about safer sex (*p* = 0.00), current CD_4_ count (*p* = 0.03), and use of substance (*p* = 0.00) had significant association with risky sexual practices.

Risky sexual practice was 2.27 times (AOR = 2.27, 95% CI: 1.02, 4.41) higher among participants who were below grade eight, 2.12 times (AOR = 2.12, 95% CI: 1.01, 5.10) higher among participants who complied high school compared to participants who had college diploma and above educational status. Married participants were engaged 2 times more in risky sexual practices (AOR = 2.07, 95% CI: 1.06, 4.02) than the unmarried.

Risky sexual practice was nearly 4 times higher (AOR = 3.74, 95% CI: 2.28, 6.13) among participants who had no concern about safer sex practice because they are on ART compared to those who had concern. Participants who had a CD_4_ count of ≥500cells/mm^3^ were engaged in risky sexual practice nearly 2 times (AOR = 1.66, 95% CI: 1.04, 2.64) more than individuals who had a CD_4_ count of less than 500cells/mm^3^. This study revealed that participants who used substances were engaged in risky sexual practice 3 times (AOR = 3.41, 95% CI: 1.83, 6.35) more than those who didn’t use substances (Table 3).

**Table 3:** Bivariate and multivariate analysis for risky sexual practice for participants who visited ART clinics of Addis Ababa public hospitals, 2017

Variables	Risky sexual practice Yes No		COR(95% CI)	AOR(95% CI)
Age				
18–23	2	10	0.5(0.093, 2.70)	0.34(0.41, 2.71)
24–29	63	90	1.75(0.77, 3.90)	1.15(0.36, 3.66)
30–35	87	113	1.93(0.88, 4.22)	1.19(0.39, 3.64)
36–41	58	104	1.39(0.63, 3.11)	1.14(0.36, 3.62)
**≥** 41	10	25	1	1
Educational status				
≤ 8	81	137	1.16(0.70–1.93)	2.27(1.01,5.10)*
9–12	107	142	1.48(0.91–2.43)	2.12(1.02,4.41)*
Diploma and above	32	63	1	1
Marital Status				
Unmarried	65	146	1	1
Married	120	68	3.96(2.61,6.02)	2.07(1.06,4.02)*
Others^a^	35	128	0.61(0.38,0.98)	2.08(0.85,5.07)
Occupation				
House wife	48	51	1	1
Daily laborer	20	68	0.31(0.17, 0.59)	0.45(0.17, 1.17)
Private job	85	118	0.77(0.47, 1.24)	0.93(0.51, 2.41)
Government employee	43	67	0.68(0.39, 1.18)	0.97(0.43, 2.23)
NGO	14	26	0.57(0.27,1.22)	0.62(0.39, 3.77)
Unemployed	10	12	0.89(0.35, 2.24)	0.80(0.50, 9.82)
Monthly family income				
< 1500	46	116	0.46(0.28, 0.74)	0.78(0.38, 1.65)
1500–2999	110	152	0.84(0.55, 1.27)	0.81(0.45, 1.45)
≥ 3000	64	74	1	1
Concern about safer sex				
Yes	99	238	1	1
No	121	104	2.79(1.96,3.97)	3.74(2.28, 6.13)*
Current CD_4_ count				
< 500cells/mm^3^	95	186	1	1
≥ 500cells/mm^3^	125	156	1.57(1.12,2.21)	1.66 (1.04, 2.64)*
Substance use				
Yes	70	35	4.09(2.61,6.42)	3.41(1.83, 6.35)*
No	150	307	1	1

^1^Note:1 = reference *p-value< 0.05 ^a^Widowed, separated, and divorced

## Discussion

Since Ethiopia is still in the midst of an HIV/AIDS epidemic, the practice of safer sex among all sexually active people, including people living with HIV, is critical to ending the problem. Although the majority of the participants reported safe sex practice, the prevalence of risky sexual practice in this study was 39.1%(95% CI: 35.2, 43.8) three months before the data collection. The finding was in line with those of other similar studies conducted at Gondar hospital, Ethiopia [10], and at Addis Ababa public hospitals, Ethiopia [6]. However, the finding in this study was higher than those of other studies conducted at Addis Ababa health centers, Ethiopia [5], India [7], and in Togo [11]. The possible reason for the variation could be study settings and comprehensive definition used in this study. The Ethiopian study was conducted at health centers, low case flow and patients’ preference of hospitals for seeking better management could decrease the prevalence. The Indian study defined risky sexual practice as inconsistent condom use with regular partners. The Togo study defined risky sexual practice as engaging in unprotected sexual intercourse with a negative or unknown sero-status partner. The prevalence of risky sexual practice was lower in this study compared to the study conducted at Felegehiwot hospital, Ethiopia [4].

In this study, educational status was inversely associated with risky sexual practice. This finding is in line with those of other studies conducted at Sokode, Togo [11] and Addis Ababa, Ethiopia [6] which showed that as the level of education increases the chance of engaging in risky sexual practices decreases.

Married participants in this study were more engaged in risky sexual practices than those who were unmarried. This might be related to their desire to conceive and have children, or some couple could think that condom use was no longer useful since they were already infected. This finding is in line with those of other studies conducted at Gondar hospital, Ethiopia [10] and Addis Ababa [5]. The finding differs from the results of other studies done at public hospitals of Addis Ababa and showed that marital status did not have any significant association with risky sexual practice [6].

Taking ART on its own does not bring good physical health unless clients are concerned about their healthy sexual practice and put in to practice what they know about safer sex [5, 6]. In this study, participants who had no concern about safer sex practice because they were on ART had significant association with risky sexual practice. Those who had no concern about safer sex practice had a higher engagement in risky sexual practice than those who had concern. This finding is in line with those of other studies conducted in India [7] and South Africa [12] which revealed that participants who were not concerned about safer sex practice due to the availability of ART were five times more likely to have had unprotected sex.

The aim of anti-retro viral therapy is to improve the quality of life and increase the life expectancy of HIV infected individuals by raising the CD_4_ count and decreasing the viral load. But following this improvement, a significant number of HIV infected individuals started to engage in risky sexual practice [5]. In this study, the CD_4_ level of individuals was inversely associated with risky sexual practice. This finding is in line with the results of another similar study conducted at Gonder hospital, Ethiopia [10] but differs from that of another study conducted in South Africa [13] which showed that the sexual behavior of individuals didn’t affect their CD_4_ count.

In this study, substance use had a significant association with risky sexual practice. Participants who used substances were engaged in risky sexual practice more than those who didn’t use substances. The findings of this study were in line with those of other studies conducted in Ethiopia [2], South Africa [13], the USA [14–17], Croatia [18], and India [19].

### Limitations of the study

- The data collection was done by nurses who were working in the ART clinics of each hospital to ensure confidentiality and privacy. Due to this, social desirability bias and interviewer bias were eminent in this study. But to decrease the bias, a training was given on the objective of the study to data collectors, and male data collectors were assigned to male respondents and females to female respondents. On top of that, data collectors gave explicit information to participants on the value of their response for the success of ART treatment and preventing PLHIV community from acquiring drug resistant strains.
- In this study, the viral loads of participants were not measured, therefore, the actual levels of HIV infectivity among participants could not be determined or reported. It was assumed that all PLHIV who engaged in risky sexual practices would put their sexual partners at risk of being infected with HIV.
- Since the issue was sensitive, some of the respondents might not have given the correct answer to concerned some of their habits.

## Conclusions

In this study, low educational status, marriage, lack of concern about safer sex practice because of being on ART, CD_4_ count ≥500cells/mm^3^, and substance use contributed to engaging in risky sexual practice.

The overall prevalence of risky sexual practice three months prior to data collection was found to be higher in this study. Unprotected sex among people living with HIV increased the risk of acquiring new viral strains that may lead to drug resistance. In the context of Ethiopia, public health efforts to promote the practice of protected sex among all sexually active people, including people living with HIV, is critical to ending the HIV/AIDS epidemic.

It is recommended that the Addis Ababa Health Bureau and ART service providers work hard on behavioral change communication. Specially, ART service providers should always advise all sexually active and married individuals to use condoms consistently and give extensive counseling to PLHIV to avoid risky sexual practice even if they have a high CD_4_ count and are on ART. In addition, health care providers need to have regular and ongoing counseling sessions with PLHIVs to avoid the use of substances. The Addis Ababa Health Bureau also has a responsibility to design strategies which support and monitor health facilities and health service providers.

## Additional file

Additional file 1: English Version of Information sheet, Consent form and questionnaire. (DOCX 27 kb)

## Abbreviations

AIDS: Acquired Immuno-deficiency Virus
AOR: Adjusted Odds Ratio
ART: Anti-retro-viral Therapy
CD4: Cell differentiation count
HIV: Human Immuno-deficiency Virus
PLHIV: People Living With HIV
SD: Standard Deviation
SPSS: Statistical package for Social Science
USA: United States of America
