# Supplementary material for: Risky sexual practice and associated factors among HIV positive adults visiting ART clinics in public hospitals in Addis Ababa city, Ethiopia: a cross sectional study
Source: BMC Public Health. 2019 Jan 28;19:113. doi: 10.1186/s12889-019-6438-5 (PMC6348678; doi:10.1186/s12889-019-6438-5)
Supplement: Supplementary file 1 — English Version of Information sheet, Consent form and questionnaire. (DOCX 27 kb) [file 12889_2019_6438_MOESM1_ESM.docx]

**Information sheet and consent form for assessment of risky sexual practice and associated factors among people living with HIV/AIDS visiting ART clinics in public hospitals in Addis Ababa city, Ethiopia.**

**University of Gondar School of Public Health**

**Name of the Principal Investigator:** Wondimagegne Belay Tadesse

**Name of the organization**: University of Gondar School of public health

**Name of the Sponsor:** Self sponsor

**Information Sheet and Consent Form prepared for participants who visit ART clinics in public hospitals in Addis Ababa city, Ethiopia.**

This information sheet and consent form is prepared by the investigator whose main aim is **to Assess Risky sexual Practice and associated factors among adult people living with HIV/AIDS, visiting ART clinics in public hospitals in Addis Ababa city .** The investigator is MPH student from University of Gondar.

**Purpose:** The purpose of this research is to measure the prevalence of Risky sexual practice and assess factors related to risky sexual practice among people living with HIV/AIDS visiting ART clinics in public hospitals in Addis Ababa city.

Sexual behavior of HIV infected people has received little attention for a range of factors. Even though many of them know about safe sexual behavior, a substantial number continue to engage in risky sexual practices that not only transmit the virus to others but also place themselves at risk of contracting secondary infection.

Therefore, the finding of this study will show the magnitude of risky sexual practice, give some hint about factors associated with it and will help policy makers to make informed decision to reduce risky sexual practice among HIV positive individuals.

**Procedure:** In order to assess risky sexual practice and related factors among people living with HIV/AIDS, We invite you to take part in our study. If you are willing to participate in our study, you need to understand and sign the consent form. Then, you will be asked to give your response by the data collectors. For this questionnaire based study, participants are HIV positive individuals visiting ART clinics in public hospitals in Addis Ababa city whose age is b/ne18-45 and who have two or more clinic visit. The responses given by the participants and the results obtained will be kept anonymous and confidential using coding system whereby no one will have access to your responses.

**Risk and/or Discomfort:** By participating in this study you may feel that it has some discomfort specially on wasting your time (**5 to 10 minutes**) but this may not be too much since you are participating for a study which will focus on a neglected issue and may bring benefit for PLWHA.

There is no risk in participating in this research project.

**Benefits:** If you participate in this study, you may not get direct benefit but your Participation is likely to help us in assessing risky sexual behavior and factors related with it, in Addis Ababa, among people living with HIV/AIDS.

**Incentives:** You will not be provided any incentives to take part in this study.

**Confidentiality and Anonymity:** The information that we will collect from this study will be kept confidential. Information about you that will be collected from the study will be Stored in a file, which will not have your name on it, but a code number assigned to it.

**Right to Refuse or Withdraw**: You have the full right to refuse from participating in this

Study (you can choose not to respond some or all of the questions) if you do not wish to

Participate; and this will not affect your health services you get at from the hospital. You have also the full right to withdraw from this study at any time you wish to, without losing any of your rights as ART clinic user of this hospital.

**Persons to contact:** If you have any question you can contact any of the following individuals and you may ask at any time you want.

1. ***Wondimagegne Belay Tadesse:*** Team leader at EPHIA project, A.A, Ethiopia

Tel: +251 114 672076/ +251 912 122184 OR +251 913 048470

E-mail: wwbelay@gmail.com OR [wondimagegneb@yahoo.com](mailto:wondimagegneb@yahoo.com)

2-***Abebaw Addis*** : University of Gondar, Gondar, Ethiopia

Tel: +251 910 905798

E-mail: abebaw.addis@gmail.com

**Consent form**

I gate full information about the study, the health professional told me that there will be no harm that will occur on me by giving the information for this questioner and the information will not disclose for anyone except for the principal investigator and also I know that I will not gate any financial support.

I confirmed that the questioner doesn’t have anything that point out/disclose my identity. Because of all this reason I decided to give information and I show my agreement with my signature.

-------------------------------------------- -----------------------------------------

Health professional signature Participant signature

Supervisor signature

**Questioner in English**

**Section 1-Socio-demographic data:** Please circle the answer of your choice

| **No.** | **Questions** | **Coding categories** | **Skip** |
| --- | --- | --- | --- |
| 101 | Sex | a-Male b-Female |  |
| 102 | Age (in years) |  |  |
| 103 | Ethnicity | a-Oromo c-Tigray  b-Amhara g-Others specify……… |  |
| 104 | Educational status | a-Illiterate f-Preparatory  b-1-4 g-College/University degree  c-5-8 h-Post graduate  d-9-10  e-10^+^ |  |
| 105 | Religion | a-Orthodox d. Catholic  b- Muslim e. Others specify…………  c-Protestant |  |
| 106 | Marital status | a-Unmarried d-Divorced  b-Married e-Widowed  c-Separated |  |
| 107 | Occupation | a-House wife d- Government employer  b-Daily laborer e-NGO  c- Private job f-unemployed |  |
| 108 | Monthly Income | a-<500 e-2000-2499  b-500-999 f-2500-2999  c-1000-1499 g-≥3000  d-1500-1999 |  |

**Section 2 -Relationship factors**

| **No.** | **Questions** | **Coding categories** | **Skip** |
| --- | --- | --- | --- |
| 201 | Are you in sexual relationship within the past 3 months? | a-Yes b-No | 202 and 203 if your answer is b |
| 202 | With how many partner/s? | a-Only one  b-More than one |  |
| 203 | Who are those partners? | a-Steady partner  b-Casual partner  c-Both with steady and casual partner |  |
| 204 | How many sexual partners did you have before testing positive? | a-Only one  b-More than one  c-I didn’t have any |  |
| 205 | How many sexual partners do you have currently? | a-Only one  b-More than one  c-I don’t have any | 206 if your answer is c |
| 206 | What type of sexual partner do you have currently? | a-Steady  b-Casual  c-Steady and casual |  |
| 207 | Do you use condom within the past 3 months? | a-Yes b-No | 208 if your answer is b |
| 208 | How often? | a-Always  b-Sometimes |  |
| 209 | What are the reasons for not using condom? | a-To get birth  b-Because it decrease sexual pleasure  c-Due to religion  d-because both of us are HIV positive  e-Partner/s don’t want to use  f-Others specify………… |  |
| 210 | Do you discuss about safe sex with your partner? | a-Yes b-No |  |
| 211 | What is your partner/s HIV Status? | a-Positive  b-Negative  c-Positive and Negative  e-Don’t know |  |
| 212 | Do you disclose your HIV status to your partner? | a-Yes b-No | 213 ,if your answer is a |
| 213 | What is the reason for not disclosing your status? | a-Not to loss acceptance from the partner  b-Fear of stigma  c-Fear of discrimination  d-Not to lose the benefit that you get  e-Other specify it…….. |  |
| 214 | Did you use condom before testing positive? | a-Yes b-No | 214 if your answer is b |
| 215 | How often? | a-Always  b-Sometimes |  |

**3-Medical related factors**

| **No.** | **Questions** | **Coding categories** | **Skip** |
| --- | --- | --- | --- |
| 301 | When do you Know that you are HIV positive? | a-3-12 month  b-13 month-24 month  c- >24 month |  |
| 302 | How do you know your HIV status at that time? | a-because you want HIV test  b-at the time you went through medical checkup.  c-because you were seriously ill and advised by your doctor to make the test.  d- due to your ANC follow up.  e-don’t remember |  |
| 303 | Do you start to use ART? | a-Yes b-No | 304 and 305 if your answer is b |
| 304 | When do you start to use ART? | a-≤12month  b-13-24 month  c->24 month |  |
| 305 | Do you concern about your safer sex practice because you are on ART? | a-Yes b-No c-I don’t know |  |
| 306 | What is your current (last check up) CD_4_ level? |  |  |
| 307 | What is your safest strategy for Safer sex practice? | a-Abstinence  b-Using condom consistently  c- Sexual exclusive relationship with only one sexual partner without condom.  d- Using condom consistently with only one sexual partner.  e-Other specify it. |  |

**4-Psyco-Social factors**

| **No.** | **Questions** | **Coding categories** | **Skip** |
| --- | --- | --- | --- |
| 401 | From the time that you know your Sero-status on wards, have you incountered one of the following?  1-Been abandoned by your spouse/partner.  2-Been excluded from a social gathering.  3-Been teased ,insulted or sworn at.  4-lost housing or not able to rent housing.  5-Lost respect/standing within the family and/or community.  6-Been given poorer quality health service.  7-Been threated with violence.  8-Been denied promotion/further training. | a-Yes b-No  a-Yes b-No  a-Yes b-No  a-Yes b-No  a-Yes b-No  a-Yes b-No  a-Yes b-No  a-Yes b-No |  |
| 402 | Within the past three months have you encountered the following conditions because you are HIV Positive?  1-Hearing people saying I would feel ashamed if I was infected with HIV.  2-Hearing people saying HIV is punishment for bad behavior.  3-Hearing people saying that promiscuous individuals are the ones that spread HIV in our community.  4- Been abandoned by your spouse/partner.  5- Been excluded from a social gathering.  6- Lost respect/standing within the family and/or community | a-Yes b-No  a-Yes b-No  a-Yes b-No  a-Yes b-No  a-Yes b-No  a-Yes b-No |  |
| 403 | Did you drink alcohol in the past three months? | a-Yes b-No | 404-408 if your answer is b |
| 404 | Did you plan your days around getthing and taking drink? | a-Never  b-Sometimes  c-Often  d-Nearly always |  |
| 405 | Did you drink alcohol in a particular way in order to increase the effect it gives you? | a-Never  b-Sometimes  c-Often  d-Nearly always |  |
| 406 | Did you fill you have to carry on drinking once you have started? | a-Never  b-Sometimes  c-Often  d-Nearly always |  |
| 407 | Is getting the effect you want more important than the particular drink you use? | a-Never  b-Sometimes  c-Often  d-Nearly always |  |
| 408 | Did you want to take more drink when the effect starts to weak off? | a-Never  b-Sometimes  c-Often  d-Nearly always |  |
| 409 | Did you take stimulants (eg. kchat, shisha, mariwana, kokiane) in the past three months? | a-Yes  b-No | 410-414 if your answer is b |
| 410 | Did you plan your days around getthing and taking stimulants? | a-Never  b-Sometimes  c-Often  d-Nearly always |  |
| 411 | Did you take stimulants in a particular way in order to increase the effect it gives you? | a-Never  b-Sometimes  c-Often  d-Nearly always |  |
| 412 | Did you fill you have to carry on taking stimulants once you have started? | a-Never  b-Sometimes  c-Often  d-Nearly always |  |
| 413 | Is getting the effect you want more important than the particular stimulant you use? | a-Never  b-Sometimes  c-Often  d-Nearly always |  |
| 414 | Did you want to take more stimulants when the effect starts to weak off? | a-Never  b-Sometimes  c-Often  d-Nearly always |  |

**5-Behavioural factors**

| **No.** | **Questions** | **Coding categories** | **Skip** |
| --- | --- | --- | --- |
| 501 | I can always manage to solve difficult problems if I try hard enough. | a-strongly disagree  b-Disagree  c-Agree  d-strongly agree |  |
| 502 | If someone opposes me, I can find the means and ways to get what I want. | a-strongly disagree  b-Disagree  c-Agree  d-strongly agree |  |
| 503 | It is easy for me to stick to my aims and accomplish my goals. | a-strongly disagree  b-Disagree  c-Agree  d-strongly agree |  |
| 504 | I am confident that I could deal efficiently with unexpected events. | a-strongly disagree  b-Disagree  c-Agree  d-strongly agree |  |
| 505 | Thanks to my resourcefulness, I know how to handle unforeseen situations. | a-strongly disagree  b-Disagree  c-Agree  d-strongly agree |  |
| 506 | I can solve most problems if I invest the necessary effort. | a-strongly disagree  b-Disagree  c-Agree  d-strongly agree |  |
| 507 | I can remain calm when facing difficulties because I can rely on my coping abilities. | a-strongly disagree  b-Disagree  c-Agree  d-strongly agree |  |
| 508 | When I am confronted with a problem, I can usually find several solutions. | a-strongly disagree  b-Disagree  c-Agree  d-strongly agree |  |
| 509 | If I am in trouble, I can usually think of a solution. | a-strongly disagree  b-Disagree  c-Agree  d-strongly agree |  |
| 510 | I can usually handle whatever comes my way. | a-strongly disagree  b-Disagree  c-Agree  d-strongly agree |  |
| 511 | I would feel embarrassed to put a condom on myself or my partner | a-strongly agree  b-Agree  c-Don’t know  d-Disagree  e-Strongly disagree |  |
| 512 | I feel confident I could gracefully remove and dispose of a condom when we have intercourse | a-strongly agree  b-Agree  c-Don’t know  d-Disagree  e-Strongly disagree |  |
| 513 | I feel confident in my ability to incorporate putting a condom on myself or my partner into foreplay | a-strongly agree  b-Agree  c-Don’t know  d-Disagree  e-Strongly disagree |  |
| 514 | I feel confident that I could use a condom with a partner without "breaking the mood." | a-strongly agree  b-Agree  c-Don’t know  d-Disagree  e-Strongly disagree |  |
| 515 | I feel confident that I could use a condom successfully | a-strongly agree  b-Agree  c-Don’t know  d-Disagree  e-Strongly disagree |  |
| 516 | I feel confident in my ability to discuss condom usage with any partner I might have | a-strongly agree  b-Agree  c-Don’t know  d-Disagree  e-Strongly disagree |  |
| 517 | I feel confident in my ability to suggest using condoms with a new partner | a-strongly agree  b-Agree  c-Don’t know  d-Disagree  e-Strongly disagree |  |
| 518 | I feel confident I could suggest using a condom without my partner feeling "diseased" | a-strongly agree  b-Agree  c-Don’t know  d-Disagree  e-Strongly disagree |  |
| 519 | I feel confident in my ability to discuss condom usage with any partner I might have | a-strongly agree  b-Agree  c-Don’t know  d-Disagree  e-Strongly disagree |  |
| 520 | I would not feel confident suggesting using condoms with a new partner because I would be afraid he or she would think I have a sexually transmitted disease | a-strongly agree  b-Agree  c-Don’t know  d-Disagree  e-Strongly disagree |  |
| 521 | I would not feel confident suggesting using condoms with a new partner because I would be afraid he or she would think I thought they had a sexually transmitted disease | a-strongly agree  b-Agree  c-Don’t know  d-Disagree  e-Strongly disagree |  |
| 522 | How do you cope difficult times in this three months? | a-acquiring social support.  b-Refraining  c-mobilizing the family to get and accept help  d-seeking spiritual support  5-passive appraisal |  |
| 523 | Who gives you great support in the past three months? | a-Family  b-Friends  c-Others specify |  |
| 524 | What type of support do you get in the past three months? | a-basic needs  b-money  c-time for self  d-time for family |  |
| 525 | What type of support do you need in the future? | a-support for growth  b-focus on improving my health status  c-to improve my physical fitness  d-intra family support.  e-child care  f-Nothing |  |
